# Untargeted Plasma Metabolomics Reveals Metabolic Alterations Associated With Idiopathic Epilepsy in Dogs

**DOI:** 10.1002/vms3.71328

**Published:** 2026-11-14

**Authors:** Piyada Na Nakorn, Boonrat Chantong, Duangthip Chatchaisak, Sataporn Phochantachinda

**Affiliations:** ^1^ Center for Research Innovation and Biomedical Informatics, Faculty of Medical Technology Mahidol University Nakhon Pathom Thailand; ^2^ Department of Pre‐clinic and Applied Animal Science, Faculty of Veterinary Science Mahidol University Nakhon Pathom Thailand; ^3^ Department of Clinical Sciences and Public Health, Faculty of Veterinary Science Mahidol University Nakhon Pathom Thailand

**Keywords:** canine epilepsy, plasma metabolomics, untargeted metabolomics

## Abstract

**Background:**

Canine idiopathic epilepsy (IE) is one of the most common chronic neurological disorders in dogs, however, its underlying pathophysiology remains incompletely understood.

**Objectives:**

This study aimed to characterise plasma metabolomic alterations associated with canine IE at two clinical points using an untargeted metabolomic approach.

**Methods:**

Plasma samples were collected from nine client‐owned healthy dogs (Normal), and nine dogs with idiopathic epilepsy evaluated at acute seizure state (Epilepsy_D0) and after a 14‐day seizure‐free interval following antiepileptic treatment (Epilepsy_D14). For metabolomic analysis, plasma from three dogs was pooled to generate three biological pools per condition. Untargeted plasma metabolomic profiling was performed using LC–QTOF‐MS in positive ionisation mode.

**Results:**

A total of 491 metabolites were retained for downstream analysis. Exploratory heatmap and principal component analyses showed differences in overall metabolic patterns among three groups. Differential abundance analysis identified distinct metabolite changes across the pairwise comparison. Pathway enrichment analysis of the significantly altered metabolites using the study‐specific metabolite set as a custom background did not identify any pathway reaching statistical significance. Six metabolites of interest, namely pyridoxal, methylimidazoleacetic acid, 2‐methylbutyroylcarnitine, indolelactic acid, homocitrulline and DL‐Normetanephrine, showed distinct abundance patterns across the study conditions.

**Conclusions:**

These findings provide preliminary evidence of plasma metabolic alterations associated with canine IE and differences between the acute seizure state and the 14‐day seizure‐free interval. Given the pooled biological design and limited effective sample size, these findings should be considered exploratory and require validation in larger cohorts using individual biological samples.

## Graphical abstract

Canine idiopathic epilepsy is associated with significant alterations in the plasma metabolome. Six metabolites, namely pyridoxal, methylimidazoleacetic acid, 2‐methylbutyroylcarnitine, indolelactic acid, homocitrulline and DL‐normetanephrine were identified as altered.

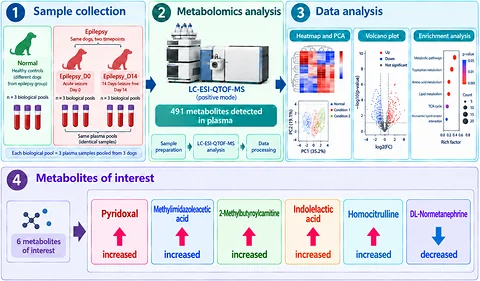

## 1 Introduction

Canine epilepsy is among the most prevalent chronic neurological disorders in dogs, with an estimated prevalence of 0.6%–0.75% in the general population (Heske et al. 2014). The International Veterinary Epilepsy Task Force (IVETF) classifies epilepsy aetiologically into structural epilepsy, reactive epileptic seizures and idiopathic epilepsy (De Risio et al. 2015). Idiopathic epilepsy (IE) is the most common cause of recurrent seizures in dogs, with a typical age of onset between 6 months and 6 years (Berendt et al. 2015). Although affected dogs may achieve a median survival time of 9.2–10.4 years (Hamamoto et al. 2016; Fredsø et al. 2014), the condition substantially reduces quality of life for both affected dogs and their owners throughout the disease course (Booth et al. 2021; Wessmann et al. 2016).

The aetiology and pathophysiology of canine IE remain incompletely understood. The principal pathophysiological mechanism involves neuronal hyperexcitability arising from an imbalance between excitatory and inhibitory neurotransmission, together with dysfunction of ion channels and neurotransmitter receptors (March 1998; Potschka et al. 2023). In addition, increasing evidence suggests that neuroinflammation and oxidative stress contribute substantially to epileptogenesis, as accumulation of inflammatory mediators and reactive oxygen species within the brain may lower seizure threshold and promote seizure progression (Eyo et al. 2017; López‐Meraz and Álvarez‐Croda 2023; Sun et al. 2019; Yuen et al. 2018; Pearson‐Smith and Patel 2017; Baka et al. 2026; Verdoodt et al. 2025).

Clinical management of canine epilepsy is complicated by the absence of specific peripheral biomarkers, rendering the diagnosis of IE fundamentally one of exclusion. According to IVETF guidelines, a Tier 1 diagnosis relies primarily on patient history and clinical presentation, whereas higher diagnostic confidence (Tier II–III) requires advanced investigations including magnetic resonance imaging (MRI), cerebrospinal fluid (CSF) analysis and electroencephalography (De Risio et al. 2015; Moore 2013). However, the high cost, limited accessibility and invasive nature of these procedures represent substantial barriers in routine veterinary practice.

Metabolomics enables broad characterisation of biological phenotypes at the small‐molecule level, providing integrated insight into metabolic processes accompanying neurological disease (Verdoodt et al. 2025; Fiehn 2002). Previous proteomics and metabolomic investigations in epileptic models have identified metabolic alterations associated with oxidative stress, neuroinflammatory pathways and amino acid metabolism, suggesting that seizure activity induces substantial systemic metabolic disturbances (Verdoodt et al. 2025; Liu et al. 2008; Baka et al. 2021). Nevertheless, metabolomic information in naturally occurring canine IE remains limited, particularly across different seizure‐related clinical conditions. Most previous studies have evaluated epileptic dogs at a single clinical time point, limiting understanding how plasma metabolomic profiles may differ between the early post‐seizure period and a subsequent seizure‐free condition.

Therefore, the present study aimed to characterise plasma metabolomic profiles in client‐owned dogs with idiopathic epilepsy during the acute seizure state and following 14 days of antiepileptic therapy, compared to healthy controls. We hypothesised that dogs with idiopathic epilepsy would exhibit different plasma metabolomic signatures associated both during acute seizure activity and following a 14‐day seizure‐free interval, and that these metabolite alterations would provide preliminary insight into metabolic processes associated with canine idiopathic epilepsy.

## 2 Materials and Methods

### 2.1 Study Population

We enrolled 18 dogs, including 9 dogs diagnosed with idiopathic epilepsy evaluated within 2 days of seizure onset at the first visit and re‐evaluated after being seizure‐free for more than 2 weeks at the second visit (acute epilepsy, D0) and a 14‐day seizure‐free interval (D14 group) and nine healthy dogs (normal group). Idiopathic epilepsy is defined as a condition in which dogs between 6 months and 6 years of age experienced their first seizure without any other underlying cause for the seizures according to basic diagnostic tools following the IVETF Tier 1 diagnostic criteria (Berendt et al. 2015). Dogs in the epilepsy group received routine veterinary treatment at the hospital.

The healthy status of the control dogs was confirmed according to their history and physical examination findings. All healthy dogs in the control group were more than 1 year old with normal vital signs and blood test results, and there were no breed or sex restrictions. Control dogs did not receive any medications other than routine monthly flea/tick/heartworm preventatives. Dogs with complications affecting neurological function, including paroxysmal dyskinesia and neoplasia, were excluded from the study. All participating dogs were housed with their owners. Blood samples were collected from dogs in all groups to determine their metabolomic profiles.

### 2.2 Blood Samples

Blood samples were collected from all dogs for haematological, biochemical and metabolomic analyses. In total, 3–5 mL of blood was drawn from either the cephalic or saphenous vein and divided into three aliquots. The first aliquot, an EDTA blood sample, was used for haematological analysis, with indices measured within 4 h of collection using an animal haematology analyser (Mindray, Shenzhen, China). The second aliquot, a heparinised sample, was centrifuged at 3500 rpm for 5 min within an hour of collection to separate the plasma. The separated plasma was used to measure clinical biochemical parameters (alanine aminotransferase, alkaline phosphatase (ALP), blood urea nitrogen, creatinine, total protein and albumin) using an automatic analyser (Beckman Coulter, Brea, CA, USA). The remaining plasma was then collected in sterile microcentrifuge tubes and stored at −80°C for subsequent metabolomic analysis.

### 2.3 Sample Preparation for Metabolomic Analysis

Equal aliquots of plasma from three individual dogs were combined to generate one pooled biological sample prior to metabolite extraction. Three pooled samples were generated for each experimental condition (Normal, Epilepsy_D0, and Epilepsy_D14). For the epilepsy group, the same three dogs contributed to the corresponding Day 0 and Day 14 pools. A 100‐µL of pooled plasma sample was mixed with three volumes of ice‐cold methanol (Wunstorfer Strasse, Seelze, Germany) and incubated at −20°C for 2 h. Subsequently, the samples were vortex‐mixed and centrifuged at 20,000 × *g* for 15 min at 4°C. The resulting supernatant was collected and dried using a nitrogen evaporator (Organomation, Berlin, MA, USA). Preparation of the dried extract was performed as previously described (Alsoud et al. 2022). Briefly, the dried extracts were then reconstituted in 250 µL of 0.1% formic acid prepared in LC–MS‐grade deionised water (CHROMASOLV, Honeywell, Wunstorfer Strasse, Seelze, Germany). The reconstituted samples were filtered through a 0.45‐µm hydrophilic nylon syringe filter prior to LC–MS/MS analysis. Subsequently, 100 µL of the prepared sample was transferred into LC glass vials containing inserts for analysis.

### 2.4 LC–MS/MS‐Based Metabolomic Profiling

Metabolite profiling was performed using electrospray ionisation quadrupole time‐of‐flight mass spectrometry (ESI‐QTOF MS), with each pooled sample analysed in triplicate technical injections, following a previously described protocol (Santajit et al. 2024) with some modifications. Chromatographic separation was achieved using a DIONEX Ultimate 3000 HPLC system (Dionex Softron, GmbH, Germering, Germany) equipped with a Zorbax Eclipse Plus C18 analytical column (2.1 × 150 mm, 1.8 µm) and a Zorbax Eclipse Plus C18 guard column (4.6 × 5 mm, 1.8 µm). The injection volume was 3 µL. The column and autosampler temperature were maintained at 40°C and 10°C, respectively. Mass spectrometric detection was carried out using a Bruker compact QTOF mass spectrometer (Bruker, Germany). MS signals were acquired over an m/z range of 50–1000 in positive ionization modes. The nebulising gas pressure, drying gas flow rate and drying gas temperature were set to 2 bars, 8 L/min and 220°C, respectively. Chromatographic separation was conducted using a binary mobile phase system comprising mobile phase A (water containing 0.1% formic acid) and mobile phase B (acetonitrile containing 0.1% formic acid). The flow rate was initially maintained at 0.25 mL/min under the following gradient conditions: 0–2 min, 99% A and 1% B; 2–17 min, linear gradient from 99% A to 1% A and 1% B to 99% B; and 17–20 min, 1% A and 99% B. Subsequently, the gradient was returned from 99% B to 99% A during 20–20.1 min, followed by equilibration at 99% A and 1% B from 20.1 to 28.5 min at a flow rate of 0.35 mL/min. Finally, the flow rate was adjusted to 0.25 mL/min from 28.5 to 30 min under the same solvent composition. Sodium formate was used as an external calibrant for mass calibration. Data acquisition consisted of two segments: an auto MS scan from 0 to 0.3 min for sodium formate calibration and an auto MS/MS acquisition from 0.3 to 30 min for metabolite fragmentation analysis. Both acquisition segments were performed in positive ionization mode at a scan rate of 12 Hz. The automatic mass scan range was set from 20 to 1300 m/z. The precursor ion isolation width was 0.5 Da, with a maximum of three precursors selected per cycle. The cycle time was set to 0.5 s with a threshold of 400 counts. Active exclusion was applied after three spectra and released after 0.2 min.

### 2.5 LC–MS/MS Data Processing and Metabolite Annotation

Raw LC‐MS/MS data were processed using MetaboScape 2022 software (Bruker, Germany). Feature extraction was performed using the T‐ReX 3D workflow with the following parameters: intensity threshold of 1000, minimum peak length of 7 spectra and peak area integration for feature quantification. Mass calibration was performed using spectra acquired during the 0.1–0.3 min interval. Auto MS/MS spectra were processed using the average method. The scan parameters included a retention time range of 0.3–25 min and a mass range of 50–1000 m/z. Metabolite identification was conducted by matching MS/MS spectra and retention times against the Bruker MetaboBase Personal Library 2.0 and the MassBank of North America (MoNA), a curated metabolomics database. Compounds with available MS/MS spectra were annotated through library matching. Finally, the processed metabolite dataset was exported in CSV format for downstream analysis. The raw LC–MS/MS data, converted mzML files, and associated metabolite annotation data have been deposited in the MetaboLights repository under accession number MTBLS15657.

### 2.6 Statistical and Metabolomic Data Analysis

#### 2.6.1 Statistical Analysis of Clinical Data

The sample size of dogs in this study was determined using the G*Power version 3.1.9.7 program (Faul et al. 2009). Data normality and homogeneity of variance were assessed using the Shapiro–Wilk and Levene's tests, respectively, and group comparisons were performed by one‐way analysis of variance (ANOVA). Statistical significance was defined as *p* < 0.05.

#### 2.6.2 Univariate Analysis of Metabolomic Data

Raw metabolite intensity values were processed prior to statistical analysis. For each pooled biological sample, technical replicate injections were summarised prior to statistical analysis. Metabolites detected in all three technical replicates were represented by the mean intensity of the three injections, whereas metabolites detected in two of three replicates were represented by the mean of the two available values. Metabolites detected in one or none of the three replicates were treated as missing values. Zero values were treated as missing values (NA). Only metabolites with complete intensity values across all samples within each comparison (comparison‐specific complete‐case dataset) were retained for downstream analysis. The datasets were log2‐transformed without additional normalisation or autoscaling. Differential metabolite abundance between groups was assessed using student's *t*‐test, with unpaired tests applied for comparisons between independent groups (Epilepsy_D0 vs. Normal and Epilepsy_D14 vs. Normal) and a paired test applied for within‐subject comparison between Epilepsy_D14 and Epilepsy_D0. To account for multiple comparisons, *p*‐values were adjusted using the Benjamini–Hochberg method to control the false discovery rate (FDR). Log2 fold change (log2FC) was calculated as the difference between mean log2‐transformed intensities of the two groups. Metabolites were considered significantly altered if they met the criteria |log2FC| ≥ 1 and FDR < 0.05. Volcano plots were generated by plotting log2FC against −log10(FDR), with significantly up‐regulated and down‐regulated metabolites highlighted using the ggplot2 package (version 4.0.2) in R software (version 4.2.1; R Foundation for Statistical Computing, Vienna, Austria). For visualisation and comparison of selected metabolites on interest across the three study conditions, pooled metabolite intensities were subjected to total intensity normalisation. The normalised intensities were compared among the Normal, Epilepsy_D0, and Epilepsy_D14 groups using ANOVA, followed by Tukey's multiple‐comparison post hoc test. Statistical significance was defined as *p* < 0.05. The normalised intensities of selected metabolites were presented as bar graphs.

#### 2.6.3 Exploratory Multivariate and Pathway Analyses

Exploratory multivariate analyses were performed using the pooled biological dataset to evaluate overall metabolic patterns among the Normal, Epilepsy_D0, and Epilepsy_D14 groups. For these exploratory analyses, missing values were imputed using a half‐minimum approach, followed by total intensity normalisation, log2 transformation, and PCA was performed to visualise the overall distribution and separation of pooled biological samples, whereas hierarchical clustering and heatmap analysis were used to examine relative metabolite abundance patterns across the three groups. These analyses were conducted in R software (version 4.2.1; R Foundation for Statistical Computing, Vienna, Austria). Pathway enrichment analysis was conducted using the MetaboAnalyst 6.0 (McGill University, Montreal, Canada). Three metabolite sets were analysed separately. First, the full set of 491 metabolites retained in the pooled dataset was used to identify metabolic pathways represented among all detected metabolites. Second, a non‐redundant set of 42 significantly altered metabolites, combined from the 57 significant entries identified across the three pairwise comparisons, was analysed against the same default reference library (Figure 3B). Third, the same 42 metabolite significant set was reanalysed using the complete list of 491 detected metabolites as a custom background set (Figure 3C). Significantly altered metabolites were identified as those with an FDR < 0.05 and |log2FC| ≥ 1.

## 3 Results

### 3.1 Haematological and Plasma Biochemical Parameters of the Study Population

Haematological and plasma biochemical parameters, including urea nitrogen (BUN), creatinine, alanine aminotransferase (ALT), glucose, albumin, globulin, total protein levels, calcium, total bilirubin, cholesterol, bile acid and ammonia showed no significant differences among the Normal and Epilepsy_D0 groups (all *p* > 0.05; Tables 1 and 2), indicating comparable routine clinical profiles across the study group. Conversely, lymphocyte count and ALP levels significantly differed between the groups (all *p* < 0.05; Tables 1 and 2).

**TABLE 1:** Haematological parameters of healthy control (the Normal: *n* = 9) and dogs with idiopathic epilepsy during the acute seizure state (Epilepsy_D0: *n* = 9).

Parameter	Reference range	Mean (± SD)		*p*‐value
		The Normal	Epilepsy_D0	
WBC	6000–17,000	12,023 (± 3309)	11,497 (± 2486)	0.708
Monocyte	180–1700	482 (± 235)	395 (± 157)	0.371
Neutrophil	3600–13,090	7893 (± 2642)	8358 (± 2264)	0.694
Lymphocyte	720–5100	3264 (± 686)	2368 (± 687)	0.014*
Eosinophil	120–1700	330 (± 349)	374 (± 392)	0.803
Basophil	0–170	1 (± 4)	1 (± 4)	0.945
Hct	35–55	47.4 (± 5.08)	47.82 (± 6.86)	0.884
Platelet	200–500	289.67 (± 102)	239.56 (± 133.9)	0.088

The asterisk (*) indicates statistical significance at *p* < 0.05

**TABLE 2:** Plasma biochemical parameters healthy control (the Normal: *n* = 9) and dogs with idiopathic epilepsy during the acute seizure state (Epilepsy_D0: *n* = 9).

Parameter	Reference range	Mean (± SD)		*p*‐value
		The Normal	Epilepsy_D0	
BUN (mg/dL)	7–27	17.89 (± 4.22)	14.89 (± 3.86)	0.135
Creatinine (mg/dL)	0.50–1.80	1.11 (± 0.10)	1.02 (± 0.19)	0.176
ALT (U/L)	10–118	83.33 (± 57.81)	101.11 (± 48.08)	0.488
ALP (U/L)	20–150	56.33 (± 37.11)	768.22 (± 633.58)	0.001*
Glucose (mg/dL)	77–125	104.44 (± 9.50)	114.22 (± 13.96)	0.102
Albumin (g/dL)	2.7–3.8	3.38 (± 0.46)	3.14 (± 0.51)	0.565
Globulin (g/dL)	2.5–4.4	2.78 (± 0.66)	3.17 (± 0.51)	0.181
Total protein (g/dL)	5.2–8.2	6.16 (± 0.56)	6.31 (± 0.95)	0.199
Calcium (mg/dL)	7.9–12.0	9.66 (± 0.61)	10.89 (± 2.55)	0.170
Total bilirubin (mg/dL)	0–0.9	0.20 (± 0.16)	0.19 (± 0.12)	0.859
Cholesterol (mg/dL)	110–320	206.78 (± 54.68)	240.78 (± 63.76)	0.242
Bile acid (µmol/L)	0–14.9	12.84 (± 11.29)	39.58 (± 58.75)	0.353
Ammonia (µmol/L)	0–98	15.22 (± 11.68)	17.56 (± 13.56)	0.756

The asterisk (*) indicates statistical significance at *p* < 0.05

### 3.2 Untargeted Metabolomics Profiling of Plasma Sample

#### 3.2.1 Global Metabolomic Profiling and Differential Metabolite Analysis

LC–MS/MS‐based untargeted metabolomic profiling was performed to investigate plasma metabolite alterations associated with idiopathic epilepsy in dogs. Metabolic analyses were conducted using three pooled biological samples for each condition (Normal, Epilepsy_D0 and Epilepsy_D14), with each pool comprising plasma from three dogs. A total of 491 metabolites were retained after data curation and used for downstream statistical and pathway analyses. Global metabolomic patterns were initially explored using heatmap visualisation, hierarchical clustering and principal component analysis (PCA). The heatmap demonstrated group‐related difference in relative metabolite abundance patterns across the pooled biological samples (Figure 1A). To further evaluate group discrimination, PCA was performed. The PCA score plot also demonstrated differences in the overall distribution of metabolomic profiles among the Normal, Epilepsy_D0 and Epilepsy_D14 groups (Figure 1B). PC1 and PC2 explained 35.2% and 19.1% of the total variance, respectively.

**FIGURE 1:**
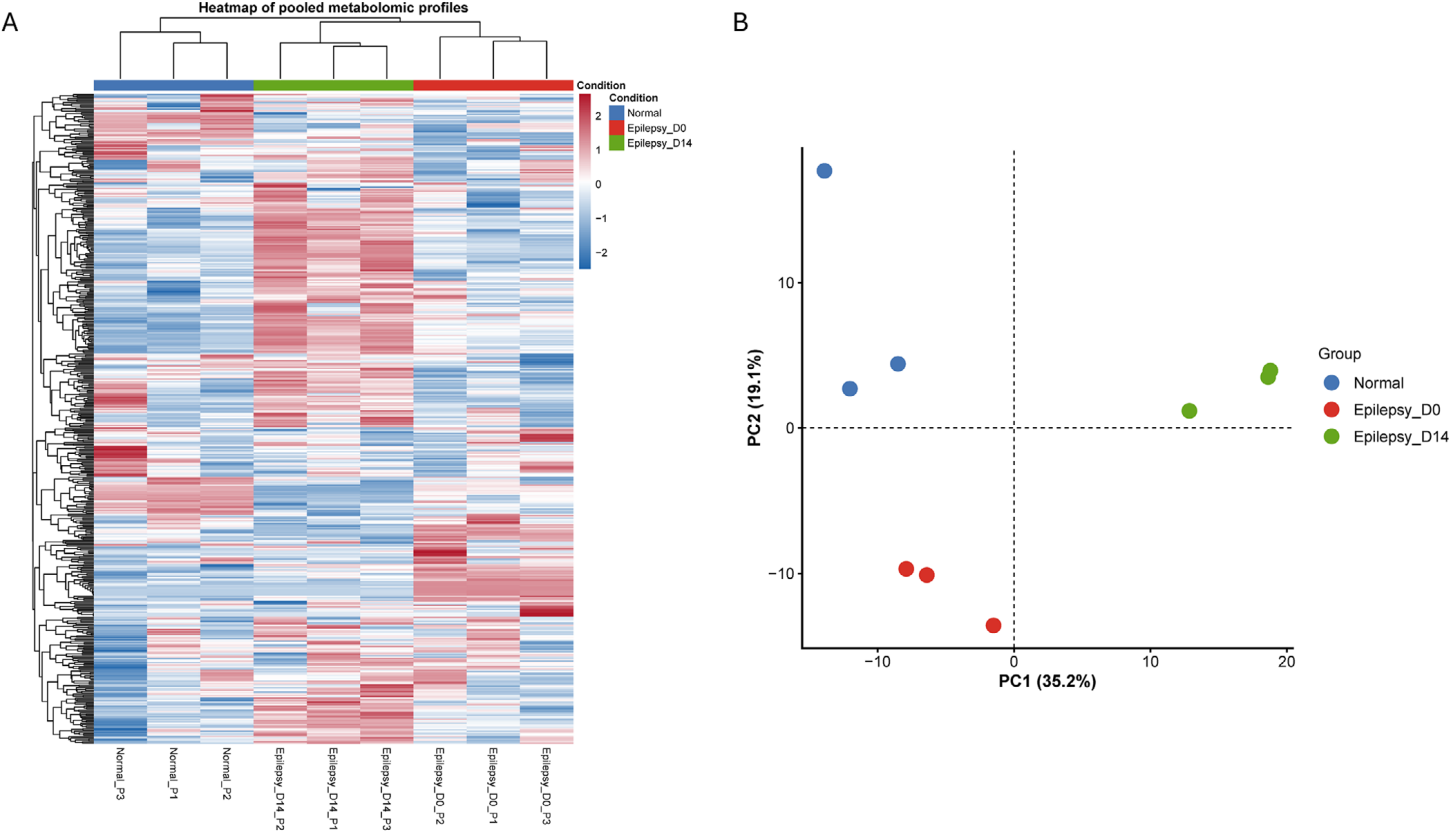
Global metabolomic profiling of pooled plasma samples from the Normal, Epilepsy_D0, and Epilepsy_D14 groups. (A) Heatmap visualization of all identified metabolites showing relative abundance patterns across study groups. Red and blue indicate relatively higher and lower metabolite abundances, respectively. (B) PCA score plot showing the overall distribution of metabolomic profiles among the three groups. Each point represents one pooled biological sample, with three pooled samples per group.

Volcano plot analysis was performed to identify differentially abundant metabolites in three pairwise comparisons using threshold of |log2FC| ≥ 1 and FDR < 0.05 (Figure 2). In the Epilepsy_D0 versus Normal comparison (Figure 2A), 19 metabolites were significantly altered, including 9 up‐regulated and 10 down‐regulated metabolites. In the paired Epilepsy_D14 versus Epilepsy_D0 (Figure 2B), 7 significantly altered metabolites were identified, including 3 up‐regulated and 4 down‐regulated metabolites. This comparison was based on corresponding pooled samples composed of the same dogs at D0 and D14. In the Epilepsy_D14 versus Normal comparison (Figure 2C), 31 metabolites were significantly altered, including 12 up‐regulated and 19 down‐regulated metabolites. Collectively, these findings demonstrate differences in plasma metabolomic profiles across the Normal, Epilepsy_D0 and Epilepsy_D14 conditions, with distinct sets of differentially abundant metabolites identified in each pairwise comparison.

**FIGURE 2:**
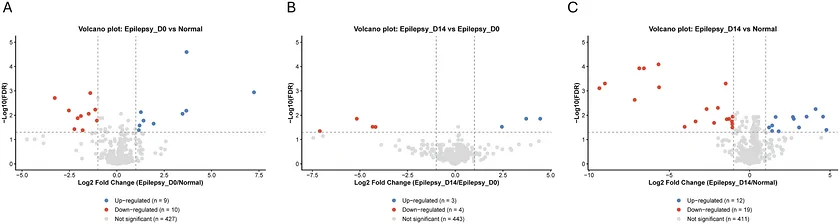
Volcano plots showing differentially abundant metabolites between (A) Epilepsy_D0 versus Normal, (B) Epilepsy_D14 versus Epilepsy_D0 and (C) Epilepsy_D14 versus Normal. Each dot represents a metabolite. The x‐axis indicates log2 fold change (log2FC), and y‐axis represents −log_10_(FDR). Metabolites with |log2FC| ≥ 1 and FDR < 0.05 were considered significantly altered. Up‐regulated metabolites are shown in blue, down‐regulated metabolites in red and non‐significant metabolites in grey.

#### 3.2.2 Pathway Enrichment Analysis

Furthermore, pathway enrichment analysis was performed to explore metabolic pathways represented in the metabolomic dataset and those associated with significantly altered metabolites. Of the 491 metabolites retained for downstream analysis, 238 were matched by MetaboAnalyst and included in the enrichment analysis. Vitamin B6 metabolism showed the strongest enrichment among the pathways represented in this full metabolite set and reached statistical significance (*p* < 0.05; Figure 3A). Other represented pathways included sphingolipid metabolism, phosphatidylcholine biosynthesis, phosphatidylethanolamine biosynthesis, tyrosine metabolism, and tryptophan metabolism. The 57 significantly altered metabolite entries identified across the three pairwise comparisons were subsequently combined, yielding 42 non‐redundant metabolites. Of these, 22 were matched by MetaboAnalyst and subjected to a separate enrichment analysis. Methylhistidine metabolism and histidine metabolism showed the strongest enrichment signals in this significantly altered set, while several pathways identified in the full metabolite analysis remained represented (Figure 3B). To account for the metabolites detectable within the present study, pathway enrichment analysis was subsequently repeated using the full list of 491 metabolites as a study‐specific custom background set, of which 238 were matched by MetaboAnalyst. Using this custom background, none of the pathways reached statistical significance (*p* < 0.05; Figure 3C). Sphingolipid metabolism, phosphatidylcholine biosynthesis and phosphatidylethanolamine biosynthesis showed the strongest enrichment signals, although none reached statistical significance. The enrichment of methylhistidine and histidine metabolism observed against the default background was not reproduced using the study‐specific background.

**FIGURE 3:**
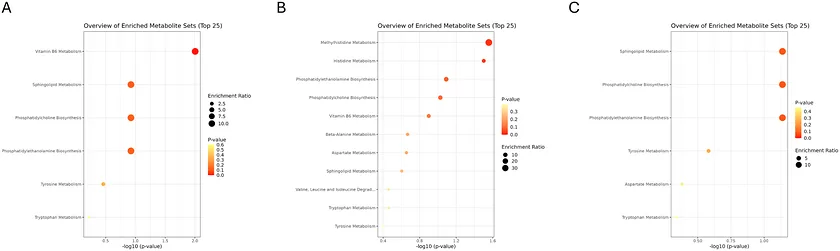
Metabolic pathway enrichment analyses of the full metabolite set and significantly altered metabolites. (A) Enrichment analysis of the full set of 491 metabolites retained for downstream analysis, of which 238 were matched in MetaboAnalyst. (B) Enrichment analysis of a non‐redundant set of 42 significantly altered metabolites identified across the three pairwise comparisons, of which 22 were matched in MetaboAnalyst. (C) Enrichment analysis of the 22 matched significantly altered metabolites using the 238 matched metabolites from the full dataset as a custom background set. Dot size represents enrichment ratio and colour intensity indicates pathway significance based on *p*‐value.

#### 3.2.3 Selected Metabolites Associated with Epilepsy‐related Metabolic Pathways

Based on the pathway enrichment results and the differential metabolite analysis, six metabolites of interest, including pyridoxal, methylimidazoleacetic acid, 2‐methylbutyroylcarnitine, indolelactic acid, homocitrulline and DL‐normetanephrine, were selected for further examination based on their differential abundance and potential biological relevance. Their relative abundance across the Normal, Epilepsy_D0 and Epilepsy_D14 groups is shown in Figure 4. Pyridoxal showed a marked increase in Epilepsy_D0 compared with both Normal and Epilepsy_D14, whereas its abundance in Epilepsy_D14 returned to a level compared to that observed in the Normal group. Methylimidazoleacetic acid and 2‐methylbutyroylcarnitine were also increased in Epilepsy_D0 relative to Normal, with lower abundance observed in Epilepsy_D14. In contrast, indolelactic acid showed a progressive increase from Normal to Epilepsy_D0 and Epilepsy_D14. Homocitrulline was highest in Epilepsy_D0 and decreased in Epilepsy_D14, while DL‐normetanephrine showed the opposite pattern, with lower abundance in Epilepsy_D0 than in Normal and a partial increase in Epilepsy_D14.

**FIGURE 4:**
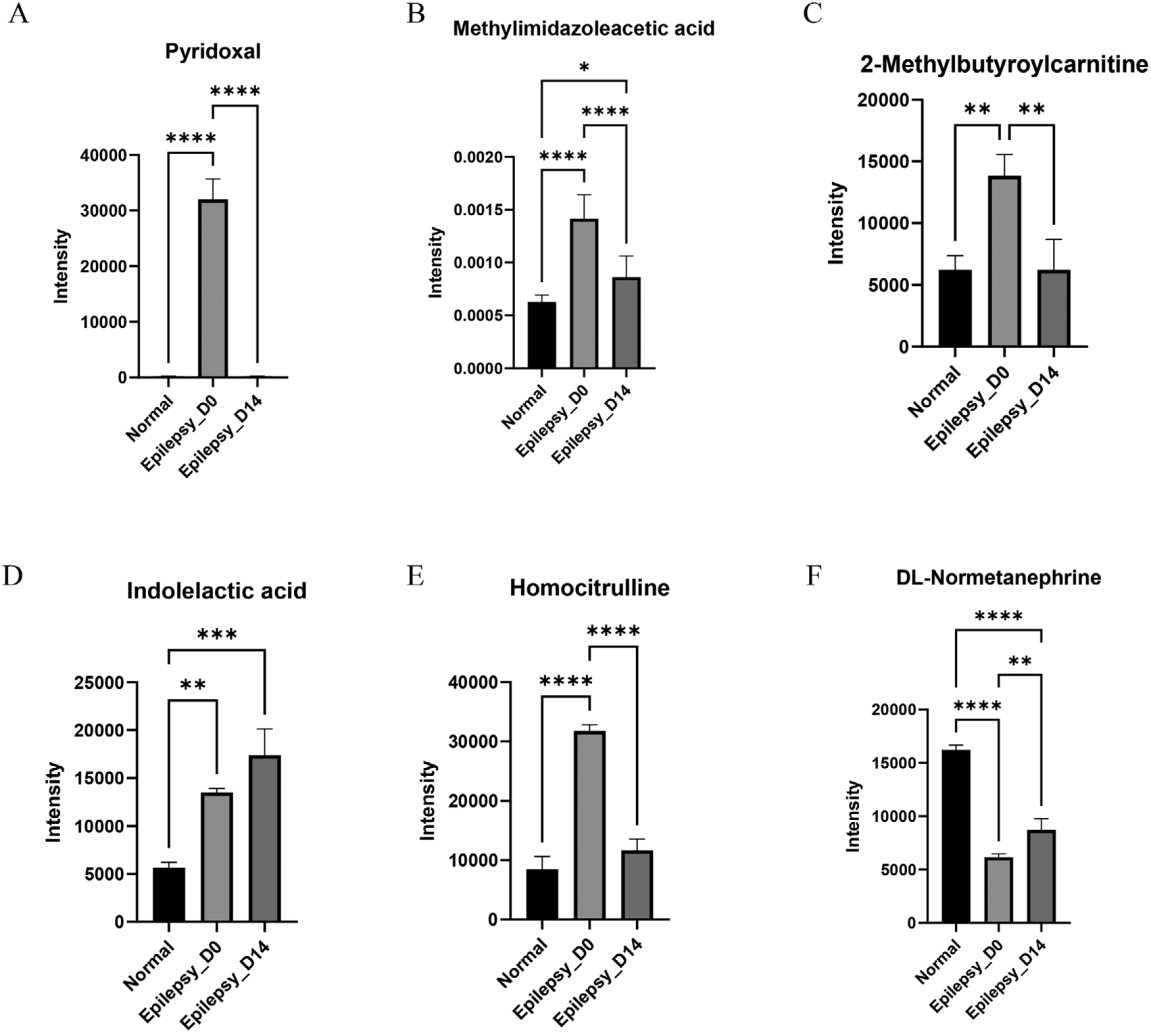
Selected metabolites associated with epilepsy‐related metabolic pathways. Relative abundance of (A) pyridoxal, (B) methylimidazoleacetic acid, (C) 2‐methylbutyroylcarnitine, (D) indolelactic acid, (E) homocitrulline and (F) DL‐normetanephrine across the Normal, Epilepsy_D0 and Epilepsy_D14 groups. Bars represent the mean ± SD.

## 4 Discussion

The present study employed untargeted plasma metabolomics to investigate metabolic alterations and metabolites associated with canine idiopathic epilepsy at two clinical time points including during the acute seizure state (Epilepsy_D0) and 14 days after seizure cessation (Epilepsy_D14). Our findings demonstrated that most routine haematological and biochemical parameters remained largely unchanged among the study groups. These findings suggest that conventional clinical parameters may not fully reflect epilepsy‐associated metabolic disturbances in dogs with idiopathic epilepsy. However, a notable exception was the significant elevation of ALP observed in the Epilepsy_D0 group. This increase may be associated with hepatic enzyme induction secondary to phenobarbital administration rather than primary hepatic injury, as other liver‐associated biochemical parameters remained within normal reference ranges (Gaskill et al. 2004; Müller et al. 2000).

Pathway enrichment analysis using the full list of 491 retained metabolites as the custom background/reference set did not identify any pathway reaching statistical significance. Nevertheless, several individual metabolites identified through differential abundance analysis showed distinct patterns across the study conditions and were considered biologically relevant. Among these, pyridoxal, a vitamin B6 vitamer, showed higher abundance in Epilepsy_D0 than in both Normal and Epilepsy_D14. Pyridoxal can be converted to pyridoxal 5'‐phosphate (PLP), the biologically active form of vitamin B6 (Ghatge et al. 2012). PLP acts as an essential cofactor for glutamate decarboxylase (GAD), which catalyses the conversion of glutamate to gamma‐aminobutyric acid (GABA), a major inhibitory neurotransmitter in the central nervous system (Parra et al. 2018; Jung et al. 2019; Ständer et al. 2025). Consistent with this, studies have shown that during a seizure, the brain triggers compensatory mechanisms, including upregulation of GAD65 and GAD67, in an attempt to increase GABA synthesis and halt seizure activity (Sperk and Pirker 2026). In our study, pyridoxal abundance was lower in Epilepsy_D14 than in Epilepsy_D0 and approached the level observed in the Normal group. The observed pyridoxal pattern may therefore be compatible with altered vitamin B6‐dependent neurotransmitter metabolism during the acute seizure state. However, the present plasma metabolomic data cannot establish whether these changes reflect central GABA metabolism or contribute directly to seizure regulation.

Methylimidazoleacetic acid is a downstream product of histamine metabolism, formed after methylation by histamine‐N‐methyltransferase and subsequent oxidative deamination by monoamine oxidase B (MAO‐B) (Granerus and Roupe 1982). Monoamines are important neuromodulator systems in the CNS and have been implicated in seizure regulation, including serotonin, noradrenaline, histamine, and melatonin (Svob Strac et al. 2016). Numerous studies on the relationship between histamine and seizures have demonstrated that elevated brain histamine can suppress seizure activity, an effect mediated in part through histaminergic modulation via activation of H1 and H3 receptors (Scherkl et al. 1991, Kim et al. 2023). This relationship between histaminergic and GABAergic signalling has also been demonstrated at the cellular level. A study in mice demonstrated that histaminergic neurons can generate a paracrine GABAergic signal by co‐releasing GABA alongside histamine (Yu et al. 2015). In our study, methylimidazoleacetic acid was increased in Epilepsy_D0 compared with Normal, which may be consistent with altered histamine metabolism during the acute seizure state. However, circulating methylimidazoleacetic acid alone cannot establish the direction or magnitude of histaminergic activity in the central nervous system. Both pyridoxal and methylimidazoleacetic acid have been reported to cross the blood‐brain barrier (Parra et al. 2018, Alghamdi et al. 2021, Khandelwal et al. 1982), providing biological plausibility for potential interactions between peripheral and central metabolism. However, the present data do not establish correspondence between plasma and brain concentrations. Taken together with the pyridoxal findings, these observations raise the possibility that metabolites related to inhibitory neurotransmitter systems may be altered during the acute seizure state, although their functional relationship to seizure activity remains to be established.

Note that 2‐methylbutyroylcarnitine (2MBC) is a naturally occurring short‐chain acylcarnitine generated during the metabolism of the branched‐chain amino acid L‐isoleucine (Xie et al. 2026). Notably, a study in human epilepsy demonstrated that isoleucine, which plays an important role in glutamatergic neurotransmission, increased above baseline before a spontaneous seizure (Ong et al. 2021). Isoleucine can subsequently be converted to 2MBC through metabolic processes that may involve the gut microbiota. A human study has reported a potential association between 2MBC and epilepsy onset and has linked 2MBC accumulation to gut microbiota dysbiosis (Xie et al. 2026; Huang et al. 2024). Similarly, canine studies have shown an association between gut dysbiosis and canine idiopathic epilepsy (Silvestrino et al. 2025; Blanquet et al. 2025).

Indolelactic acid is a tryptophan‐derived metabolite produced mainly by gut bacteria (Meng et al. 2020). It can activate the aryl hydrocarbon receptor and has been reported to suppress neuroinflammatory signalling through inhibition of the NLRP3/NF‐κB pathway, with resulting reductions in inflammatory cytokines such as IL‐1β, IL‐6, and IL‐18. Indolelactic acid has also been reported to inhibit acetylcholinesterase (AChE) activity and reduce microglial activation (Qian et al. 2024; Qin et al. 2025). In mice, it has also shown neuroprotective effects, including reductions in soluble Aβ levels (Kim et al. 2024).

Both 2‐methylbutyroylcarnitine and indolelactic acid showed altered abundance across the study conditions. The compound 2‐methylbutyroylcarnitine was increased in Epilepsy_D0 and decreased in Epilepsy_D14 toward the level observed in Normal, whereas indolelactic acid showed higher abundance in both epilepsy conditions, with the highest level observed in Epilepsy_D14. Because both metabolites have previously been linked to host‐microbial metabolic processes, these findings raise the possibility of altered gut microbiota‐associated metabolism in canine epilepsy. However, microbiome composition was not assessed in the present study, and a direct gut‐brain axis mechanism cannot be inferred from these metabolomic data alone.

Homocitrulline is a non‐proteinogenic amino acid generated through the non‐enzymatic carbamylation of lysine residues as a post‐translational modification product (Turunen et al. 2014). Under physiological conditions, homocitrulline is present at low concentrations in biological fluids as a minor by‐product of urea cycle activity (Gerritsen et al. 1962). In the present study, plasma homocitrulline abundance was higher in Epilepsy_D0 compared with Normal, and was no longer significantly different from Normal by Epilepsy_D14. Elevated homocitrulline concentrations have previously been reported in hyperornithinemia–hyperammonemia–homocitrullinuria (HHH) syndrome, a metabolic disorder associated with neurological dysfunction and seizures. (Nguyen et al. 2024). Although the present findings do not indicate HHH syndrome, the observed elevation of homocitrulline may be consistent with alterations in nitrogen‐related metabolism, however, the biological significance of this observation in canine epilepsy remains uncertain.

Normetanephrine is an inactive metabolite of the catecholamine norepinephrine (Eisenhofer et al. 1995). Animal studies suggest that norepinephrine can modulate seizure activity, with antiepileptic effects on the limbic system mediated both by preventing seizure development and by restoring normal brain circuitry function (Berger et al. 2021; Biagioni et al. 2023). Moreover, a study in mice showed that norepinephrine deficiency increases seizure susceptibility (Szot et al. 1999; Ahern et al. 2006). Consistent with this anticonvulsant role, normetanephrine showed lower abundance in Epilepsy_D0 than in Normal and increased in Epilepsy_D14 relative to D0, although its abundance remained lower than that observed in Normal. This pattern suggests altered peripheral catecholamine‐related metabolism across the study conditions. However, changes in circulating normetanephrine cannot be directly interpreted as changes in central noradrenergic activity.

Collectively, the metabolomic findings indicate differences in several metabolic processes across the Normal, Epilepsy_D0 and Epilepsy_D14 conditions, including metabolites related to vitamin B6 metabolism, amino acid metabolism, lipid‐related metabolism and neurotransmitter‐associated pathways. Some metabolite alterations observed during the acute seizure state were attenuated after the 14‐day seizure‐free interval, whereas others persisted or showed different patterns across the two epilepsy conditions. These observations suggest that the plasma metabolomic profiles differed between the acute seizure state and the 14‐day seizure‐free interval. However, the present pooled‐sample design does not allow determination of individual metabolic trajectories, causal mechanisms or treatment‐specific effects.

Several limitations of the present study should be acknowledged. First, although plasma samples were obtained from nine healthy dogs and nine dogs with idiopathic epilepsy, with the latter sampled during the acute seizure condition (D0) and again after a 14‐day seizure‐free interval (D14), metabolomic analysis was performed using three pooled biological samples per condition, each comprising plasma from three dogs. Pooling reduced the effective biological sample size and obscured inter‐individual variability, thereby limiting statistical power and generalisability. Therefore, the findings should be considered exploratory and require confirmation using individually analysed samples in larger, independent cohorts. Second, although idiopathic epilepsy was diagnosed according to the IVETF Tier I consensus criteria, MRI and electroencephalographic (EEG) confirmation were not available for all enrolled animals. Third, additional multi‐omics approaches are required to validate the present findings and further elucidate the molecular mechanisms underlying canine idiopathic epilepsy. Furthermore, pathway enrichment results should be interpreted cautiously. Of the 491 metabolites retained for analysis, 238 were matched in MetaboAnalyst, whereas only 22 of the 42 non‐redundant significantly altered metabolites were matched in the corresponding enrichment analysis. Consequently, incomplete metabolite mapping and the relatively small number of matched metabolites in the significantly altered set may have influenced pathway representation.

In conclusion, this exploratory untargeted metabolomic study identified differences in plasma metabolomic profiles among the Normal, Epilepsy_D0 and Epilepsy_D14 conditions. Although no metabolic pathway reached statistical significance when the study specific set was used as the custom background, several individual metabolites, including pyridoxal, methylimidazoleacetic acid, 2‐methylbutyroylcarnitine, indolelactic acid, homocitrulline and DL‐Normetanephrine showed distinct abundance patterns across the study conditions. These findings provide preliminary evidence of metabolic alterations associated with canine idiopathic epilepsy and differences between the acute seizure state and the 14‐day seizure‐free interval. Given the pooled biological design and limited effective sample size, these observations should be considered hypothesis‐generating and require validation in larger studies using individual biological samples before their mechanistic or clinical relevance can be established.

## Author Contributions

**Piyada Na Nakorn**: conceptualization, methodology, validation, formal analysis, investigation, writing – original draft, writing – review and editing, data curation, visualization. **Boonrat Chantong**: conceptualization, writing – review and editing, methodology, formal analysis, data curation, investigation. **Duangthip Chatchaisak**: conceptualization, methodology, formal analysis, writing – review and editing, investigation, resources, data curation, writing – original draft, visualization. **Sataporn Phochantachinda**: writing – original draft, writing – review and editing, conceptualization, methodology, validation, funding acquisition, formal analysis, investigation, resources, supervision, project administration. All authors have read and agreed to the published version of the manuscript.

## Funding

This research project has been supported by Mahidol University (Fundamental Fund: fiscal year 2025 by National Science Research and Innovation Fund), Project number FF‐170/2568.

## Ethics Statement

The study was approved by the Committee on the Care and Use of Laboratory Animals in the Faculty of Veterinary Science, Mahidol University (Salaya, Thailand; approval number: MUVS‐2024‐08‐51). All experiments adhered to relevant guidelines and regulations. Prior to enrollment, written informed consent was obtained from all dog owners.

## Conflicts of Interest

The authors declare no conflicts of interest.

## Data Availability

The metabolomics dataset has been deposited in the MetaboLights repository under accession number MTBLS15657. All data included in this work are available upon request by contact with the corresponding author.
